# 2203. Involvement of Respiratory Viruses in Community-Acquired Alveolar Pneumonia (CAAP) in Children < 5 Years in Southern Israel, Before and During the COVID-19 Pandemic

**DOI:** 10.1093/ofid/ofac492.1822

**Published:** 2022-12-15

**Authors:** Dana Danino, Bert Adriaan van der Beek, Shalom Ben-Shimol, Yonat Shemer Avni, David Greenberg, Ron Dagan

**Affiliations:** Soroka University Medical Center, Pediatric Infectious Disease Unit, Beer Sheva, HaDarom, Israel; Ben Gurion Univerisity, Beer Sheva, HaDarom, Israel; Soroka University Medical Center, Beer Sheva, HaDarom, Israel; Soroka Medical Center, Beer Sheva, HaDarom, Israel; Soroka University Medical Center, Beer Sheva, HaDarom, Israel; Ben-Gurion University of the Negev, Beer Sheva, HaDarom, Israel

## Abstract

**Background:**

Respiratory syncytial virus (RSV) and human metapneumovirus (hMPV) and to a lesser extent, parainfluenza and influenza viruses have been associated with pneumonia in young children. In contrast, adenovirus (AdV) and rhinovirus (RhV) are usually not associated with pneumonia. We aimed to evaluate the involvement of the four pneumonia associated viruses (grouped as PAV) in pediatric CAAP, before and during the COVID-19 pandemic.

**Methods:**

CAAP incidence and viral activity surveillance in southern Israel in children < 5y and virological detection methods were described previously (Danino. *CID* 2022, https://doi.org/10.1093/cid/ciab1014). We reviewed the period of Jan 2016 - Mar 2022. Most cases of COVID-19 in children < 5y occurred during Dec 2021 - Mar 2022 (**Figure 1**); Over 95% of children admitted for respiratory disease were tested for COVID-19 (PCR). Since AdV and RhV activity was previously not associated with CAAP and tended to be equally involved in mixed and single infections, the current analysis was done for the four PAV only.

**Results:**

CAAP incidence dynamics closely resembled the four PAV (grouped) activity dynamics (**Figure 2A, 2B**) with very low activity during the expected peak in winter 2020-2021, but with an off-season resurgence from spring 2021. Even though most CAAP episodes during the pandemic coincided with peak COVID-19 rates, only 9 CAAP episodes were COVID-19 positive (7 in 2022, of which 5 were RSV positive). Out of 3,430 CAAP episodes 55% were tested for PAV, of which 61% were positive, with similar rates before and during the pandemic. RSV was the most common involved virus, followed by hMPV. The virus distribution in CAAP during the entire period reflected their activity in the community **(Figure 2C)**. Unlike pre-pandemic years where all four PAV appeared almost simultaneously, in 2021 PAV resurged sequentially, resulting in successive involvement in CAAP episodes, suggesting a causative association.

**Figure:**
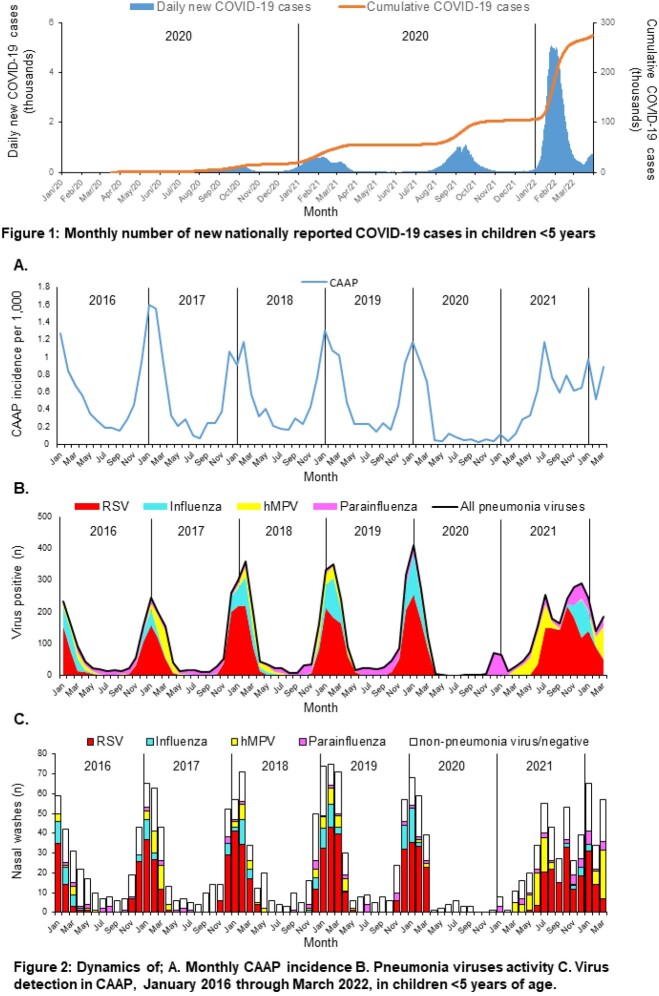

**Conclusion:**

1. SARS-CoV-2 was only rarely involved in CAAP in young children. 2. PAV were involved in 61% of CAAP episodes in children < 5y with predominance of RSV and hMPV. 3. The atypical dynamics imposed by the COVID-19 pandemic suggests a causative association between PAV and CAAP.

**Disclosures:**

**All Authors**: No reported disclosures.

